# The V-Localizer for Stereotactic Guidance

**DOI:** 10.7759/cureus.16535

**Published:** 2021-07-21

**Authors:** Russell A Brown, Armando L Alaminos-Bouza, Andres E Bruna, Mark Sedrak

**Affiliations:** 1 Principal Engineer, Retired, Palo Alto, USA; 2 Medical Physics, MEVIS Informática Médica Ltda., São Paulo, BRA; 3 Medical Physics, Fi.Me. Fïsica Médica Srl, Córdoba, ARG; 4 Neurosurgery, Kaiser Permanente, Redwood City, USA

**Keywords:** stereotactic surgery, image-guided radiosurgery, magnetic resonance imaging (mri), image-guided surgery, frame-based stereotactic surgery, stereotactic frame, n-localizer, computed tomography (ct), stereotactic radiosurgery (srs), monte carlo simulation

## Abstract

Image-guidance for frame-based stereotaxis is facilitated by incorporating three to four N-localizers or Sturm-Pastyr localizers into a stereotactic frame. An extant frame that incorporates only two N-localizers violates the fundamental principle of the N-localizer, which requires three non-colinear points to define a plane in three-dimensional space. Hence, this two N-localizer configuration is susceptible to error. The present article proposes the V-localizer that comprises multiple diagonal bars to provide four or more non-colinear points to minimize error.

## Introduction

Image-guidance for frame-based stereotaxis is facilitated by incorporating three to four N-localizers or Sturm-Pastyr localizers into a stereotactic frame [1,2]. Monte Carlo (MC) simulation [3] predicts that the attachment of one N-localizer to each of the anterior, posterior, and left and right lateral faces of a rectangular stereotactic frame affords the highest accuracy [4]. To avoid contact with the computed tomography (CT) scanner couch, the posterior N-localizer is often omitted, which results in a configuration that comprises three N-localizers [4]. Moreover, to promote patient comfort and minimize claustrophobia, the omission of both the anterior and posterior N-localizers results in a configuration that includes only two N-localizers [5]. For this two N-localizer configuration, one N-localizer is attached to each of the left and right lateral faces of a rectangular frame. However, this configuration provides insufficient information to determine the orientation of a CT image plane in three-dimensional (3D) space. Although each of the two N-localizers defines the $\left(x, y, z\right)$ coordinates of a point where the CT image plane intersects that N-localizer, three points of intersection are required to determine the 3D spatial orientation of the CT image plane. Hence, incorporation of only two N-localizers into a stereotactic frame violates the fundamental principle of the N-localizer, which requires that each CT image provide all information necessary to determine the 3D spatial orientation of the CT image plane [6].

## Technical report

Overview of the V-localizer

**Figure 1:**
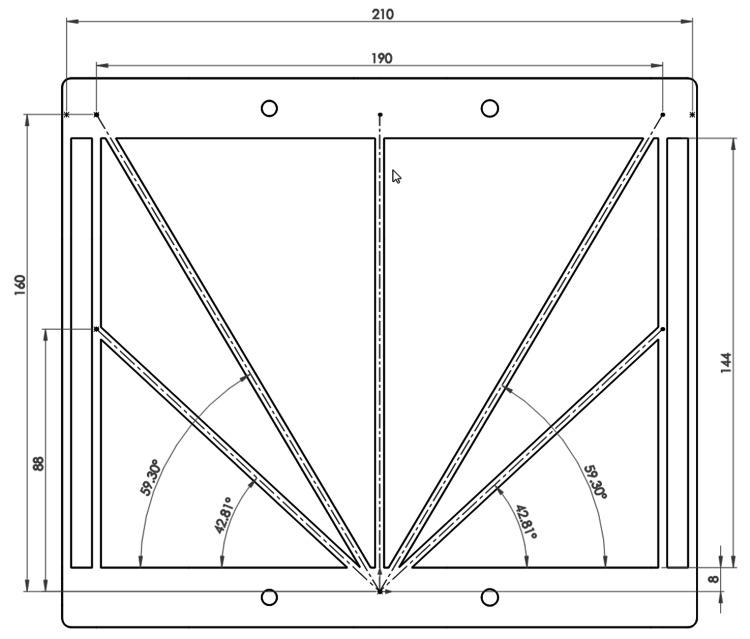
V-Localizer All dimensions are specified in millimeters (mm). The two diagonal bars that are angled at $42.81^{\large\circ}$ abut the two vertical bars that are separated by 190mm.

To enable the incorporation of only two localizers, wherein one localizer is attached to each lateral face of a rectangular stereotactic frame, several localizers that comprise two or more diagonal bars (instead of only one diagonal bar) have been proposed recently [7]. The most promising of these localizers are the M-localizer and Z-localizer. The present article proposes the V-localizer that combines elements from these two localizers, as shown in Figure 1. The two diagonal bars that are angled at $42.81^{\large\circ}$ and the two vertical bars that are separated by 210mm are elements from the Z-localizer. The two diagonal bars that are angled at $59.30^{\large\circ}$, the two vertical bars that are separated by 190mm, and the central vertical bar are elements from the M-localizer.

**Figure 2:**
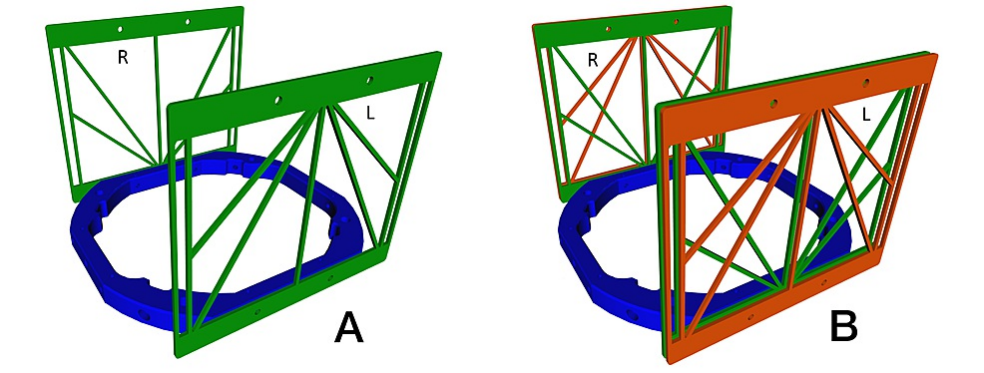
Two V-Localizers and Four V-Localizers Affixed to Stereotactic Frames (A) One V-localizer is affixed to each lateral face of a stereotactic frame. Each V-localizer is inverted top-to-bottom relative to the opposite V-localizer, similar to the configuration proposed for two M-localizers [7]. (B) Two V-localizers are affixed to each lateral face of a stereotactic frame. One V-localizer (shown in red) is inverted top-to-bottom relative to the adjacent V-localizer (shown in green). R: right; L: left

Figure 2 depicts two proposed configurations for affixing V-localizers to a rectangular stereotactic frame. The configuration depicted in Figure 2A affixes two V-localizers to a stereotactic frame. In this configuration, one V-localizer is affixed to each lateral face of the frame. The configuration depicted in Figure 2B affixes four V-localizers to a stereotactic frame. In this configuration, two V-localizers are affixed to each lateral face of the frame.

**Figure 3:**
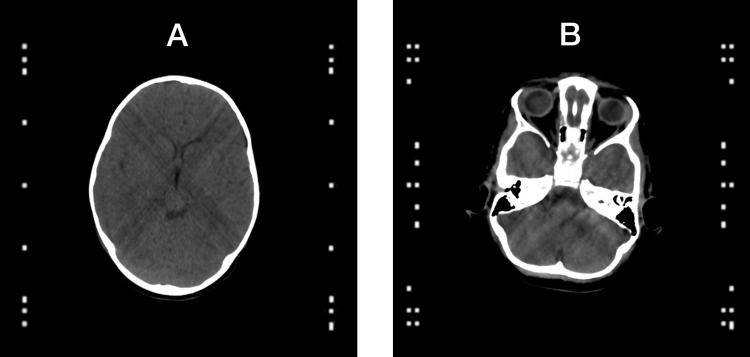
Simulated CT Images Created by the Two V-Localizer and Four V-Localizer Configurations (A) A CT scan slice positioned at height $z=80$mm relative to the two V-localizer configuration shown in Figure 2A creates one column of fiducials along each of the left and right edges of a CT image. (B) A CT scan slice positioned at height $z=15$mm relative to the four V-localizer configuration shown in Figure 2B creates two columns of fiducials along each of the left and right edges of a CT image.

Figure 3 presents simulated CT images created by the proposed V-localizer configurations shown in Figure 2. The CT image presented in Figure 3A is created by a CT scan slice positioned at height $z=80$mm relative to the two V-localizer configuration shown in Figure 2A. The CT image presented in Figure 3B is created by a CT scan slice positioned at height $z=15$mm relative to the four V-localizer configuration shown in Figure 2B. The quadrilaterals near the left and right edges of both CT images are fiducials that are created by the intersection of the CT scan slice with the vertical and diagonal bars of the V-localizer. Each fiducial is either a square, a short rectangle, or an elongated rectangle, depending on whether the CT scan slice intersects a vertical bar, a $59.30^{\large\circ}$ diagonal bar, or a $42.81^{\large\circ}$ diagonal bar, respectively. Fiducials facilitate transformation of the $\left(u, v\right)$ coordinates of a target point defined in the two-dimensional (2D) coordinate system of the CT image into $\left(x, y, z\right)$ coordinates in the three-dimensional (3D) coordinate system of the stereotactic frame [8]. Fiducials are discussed in detail in the Appendices.

Monte Carlo simulation

This article reports root mean square errors (RMSe) calculated via MC simulation for the two and four V-localizer configurations. These RMSe are compared to the RMSe for the M-localizer, the Z-localizer, a four N-localizer configuration, and the Brown-Roberts-Wells (BRW) localizer that embodies a three N-localizer configuration [1,4,7]. MC simulation models random fluctuations of the $\left(u, v\right)$ coordinates of the centroids of fiducials in a CT image. These fluctuations produce random perturbations of the $\left(x, y, z\right)$ coordinates of target points whose $\left(u, v\right)$ coordinates are transformed from the 2D coordinate system of the CT image into the 3D coordinate system of a stereotactic frame to obtain perturbed $\left(x, y, z\right)$ coordinates. For each type of localizer, MC calculates the RMSe via millions of iterations. For each iteration: (1) the unperturbed $\left(u,v\right)$ coordinates of the centroids of fiducials in the CT image are used to transform a target point from 2D to 3D in order to obtain the $\left(x, y, z\right)$ coordinates of an unperturbed target point; (2) the unperturbed $\left(u,v\right)$ coordinates of the centroids of fiducials in the CT image are perturbed randomly and then used to transform the target point from 2D to 3D in order to obtain the $\left(x,y,z\right)$ coordinates of a perturbed target point; and (3) the square of the 3D Euclidean distance between the unperturbed target point and the perturbed target point is summed. After millions of iterations, the RMSe is calculated from the sum.

The transformation of a target point from 2D to 3D is facilitated by fiducials; for example, the fiducials depicted in Figure 3. This transformation is accomplished by a 3-by-3 matrix that is computed from the $\left(x, y, z\right)$ coordinates of three points (each of which lies on the long axis of a diagonal bar) together with the $\left(u, v\right)$ coordinates of the centroids of three corresponding fiducials created by the respective diagonal bars [8]. This approach may be extended to improve the RMSe by defining an overdetermined system of linear equations, the solution to which is the 3-by-3 transformation matrix [4]. The overdetermined system is defined by: (1) the $\left(x ,y, z\right)$ coordinates of a point that lies on the long axis of each of at least three diagonal bars, (2) the $\left(x, y\right)$ coordinates of a point that lies on the long axis of each of numerous vertical bars, and (3) the $\left(u, v\right)$ coordinates of the centroids of the corresponding fiducials created by the respective diagonal and vertical bars. Table 1 shows the number of diagonal and vertical bars, and hence the number of fiducials, that define the overdetermined systems of linear equations for the M-localizer, Z-localizer, BRW localizer, four N-localizer configuration, and two and four V-localizer configurations.

**Table 1:** Numbers of Diagonal and Vertical Bars That Define the Overdetermined Systems of Linear Equations for Various Localizers The numbers of diagonal and vertical bars are shown for three N-localizers, four N-localizers, two M-localizers, two V-localizers, two Z-localizers, and four V-localizers. BRW: Brown-Roberts-Wells

Localizer	Diagonal Bars	Vertical Bars
BRW (Three N)	3	6
Four N	4	8
Two M	4	6
Two V	8	6
Two Z	14	4
Four V	16	12

MC simulation randomly perturbs the $\left(u,v\right)$ coordinates of the centroids of the fiducials. The maximum magnitude of the random perturbations for each type of localizer is directly proportional to the field of view (FOV) of the CT image. The FOV must be sufficiently large that the fiducials created by the diagonal and vertical bars are all visible in the CT image. Hence, the FOV approximately equals the perpendicular distance from the center of the stereotactic frame to the farthest vertical bar, increased somewhat to account for a tilted CT image plane wherein the non-perpendicular distance to the farthest vertical bar is increased. A constant image resolution of 512x512 pixels spreads the pixels across the FOV; consequently, the nominal pixel size and the maximum magnitude of the random perturbations are directly proportional to the FOV. Table 2 shows the FOV, the nominal pixel size for a 512x512 image, and the maximum magnitude of the perturbations for each type of localizer, assuming a 1mm maximum magnitude for both the BRW localizer and the four N-localizer configuration that have the smallest nominal pixel size.

**Table 2:** Field of View, Nominal Pixel Size, and Maximum Magnitude of the Perturbations for Various Localizers The nominal pixel size and the maximum magnitude of the perturbations are directly proportional to the FOV. The maximum magnitude of the perturbations is chosen to be 1mm for both the BRW localizer and the four N-localizer configuration. All other magnitudes scale proportionally. BRW: Brown-Roberts-Wells; FOV: field of view

Localizer	FOV (mm)	Pixel Size (mm)	Perturbation (mm)
BRW (Three N)	284.0	0.5547	1.000
Four N	284.0	0.5547	1.000
Two M	305.5	0.5967	1.076
Two Z	313.3	0.6119	1.103
Two V	317.2	0.6196	1.117
Four V	332.4	0.6492	1.170

For each type of localizer, MC simulation performs $2^{21}\approx 2$ million iterations at each of numerous heights $z$, where $z$ is incremented by 2mm throughout the vertical extent of the localizer. At each height, unperturbed and randomly perturbed centroids of fiducials are used to construct unperturbed and perturbed 3-by-3 transformation matrices that transform five target points from the $\left(u,v\right)$ coordinate system of the CT image into the $\left(x,y,z\right)$ coordinate system of the stereotactic frame to obtain unperturbed and perturbed $\left(x,y,z\right)$ coordinates, respectively, for each target point. The target points, whose $\left(u,v\right)$ coordinates are expressed in mm relative to the center of the CT image, are located at center $\left(0,0\right)$; right lateral $\left(+50, 0\right)$; left lateral $\left(-50,0\right)$; anterior $\left(0,+50\right)$; posterior $\left(0,-50\right)$; and anterolateral $\left(+50,+50\right)$. For each iteration and each target point at each height, the squared 3D Euclidean distance between the unperturbed and perturbed target point is summed. After 2 million iterations, the RMSe is calculated from the sum for each target point. The results of the MC simulation are presented below.

## Discussion

**Figure 4:**
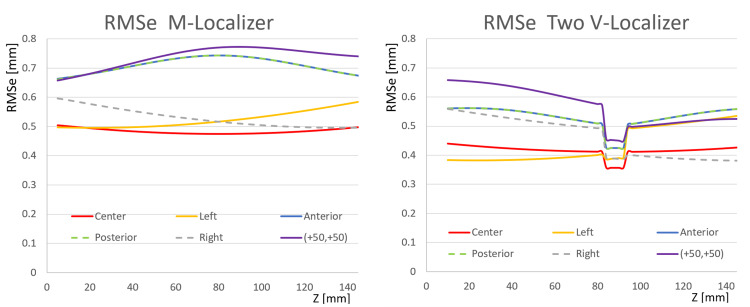
Plots of RMSe vs. Height $\left(Z\right)$ for the M-Localizer and the Two V-Localizer Configuration The RMSe of the M-localizer exceeds the RMSe of the two V-localizer configuration for each target point at all heights $\left(z\right)$. For both localizers, the plots for anterior and posterior targets are superimposed. The discontinuities of infinite slope in the plots for the V-localizer occur at heights where a diagonal bar abuts against a vertical bar. RMSe: root mean square errors

Figure 4 compares the RMSe of the M-localizer to the RMSe of the two V-localizer configuration shown in Figure 2A. For each target point, the RMSe of the M-localizer exceeds the RMSe of the V-localizer for four reasons. First, Table 1 shows that the V-localizer comprises four more diagonal bars than the M-localizer. Hence, the larger number of fiducials that define the overdetermined system of linear equations for the V-localizer decrease its RMSe compared to the RMSe of the M-localizer [4]. Second, the four additional bars of the V-localizer are the diagonal bars that provide $\left(x, y, z\right)$ coordinates to the overdetermined system of linear equations, as opposed to vertical bars that provide only $\left(x, y\right)$ coordinates that do not decrease the error in $z$. Third, Figure 1 demonstrates that the V-localizer includes two low-slope $\left(42.81^{\large\circ}\right)$ diagonal bars. The error in the $z$-coordinate provided by a diagonal bar is proportional to the slope of that bar [4]; hence, low-slope diagonal bars produce less error than high-slope diagonal bars. Therefore, the low-slope diagonal bars of the V-localizer decrease its RMSe compared to the RMSe of the M-localizer that includes only high-slope $\left(59.30^{\large\circ}\right)$ diagonal bars [7]. Fourth, in some CT images, the low-slope diagonal bars of the V-localizer extend farther anteriorly and posteriorly than any diagonal bars of the M-localizer, as discussed in the Appendices. Hence, these low-slope diagonal bars increase the area of the polygon enclosed by fiducials in the CT image and decrease the RMSe of the V-localizer compared to the RMSe of the M-localizer [4,7,9,10].

The low-slope diagonal bars of the V-localizer abut against vertical bars, as shown in Figure 1. This abutment complicates interpretation of the fiducial pattern for the V-localizer because the $\left(u, v\right)$ and $\left(x, y, z\right)$ coordinates associated with a fiducial that is created by an abutting diagonal bar must be excluded from the calculation of the 3-by-3 transformation matrix, as discussed in the Appendices. Evidence for this exclusion appears in Figure 4 as discontinuities of infinite slope in the RMSe plots for the V-localizer. These discontinuities occur at heights where a diagonal bar abuts against a vertical bar, thus excluding the diagonal bar from contributing to the calculation of the transformation matrix and hence increasing the RMSe in a stepwise manner.

**Figure 5:**
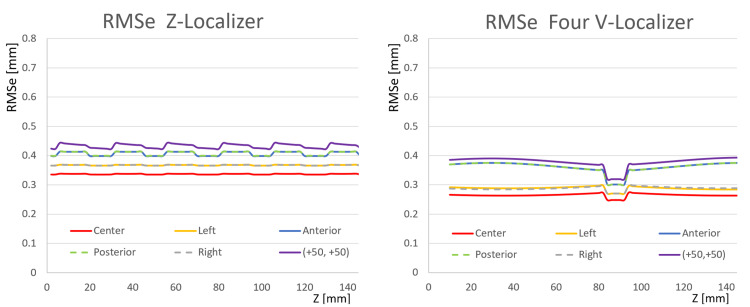
Plots of RMSe vs. Height $\left(Z\right)$ for the Z-Localizer and the Four V-Localizer Configuration The RMSe of the Z-localizer exceeds the RMSe of the four V-localizer configuration for each target point at all heights $\left(z\right)$. For both localizers, the plots for anterior and posterior targets are superimposed and the plots for left and right targets are superimposed. The discontinuities of infinite slope, which are more prominent in the plots for the V-localizer than in the plots for the Z-localizer, occur at heights where a diagonal bar abuts against a vertical bar. RMSe: root mean square errors

Figure 5 compares the RMSe of the Z-localizer to the RMSe of the four V-localizer configuration shown in Figure 2B. For each target point, the RMSe of the Z-localizer exceeds the RMSe of the V-localizer for three reasons. First, Table 1 shows that the V-localizer comprises two more diagonal bars and eight more vertical bars than the Z-localizer. Hence, the larger number of fiducials that define the overdetermined system of linear equations for the V-localizer decrease its RMSe compared to the RMSe of the Z-localizer [4]. Second, two of the additional bars are diagonal bars that provide $\left(x, y, z\right)$ coordinates to the overdetermined system of linear equations, as opposed to vertical bars that provide only $\left(x, y\right)$ coordinates that do not decrease the error in $z$. Third, Figure 3B shows that the four V-localizer configuration apportions the fiducials into four colinear sets, in contrast to two colinear sets for the Z-localizer. The four sets position fiducials farther either anteriorly or posteriorly in a particular CT image for the V-localizer than for the Z-localizer, as discussed in the Appendices. Because these anterior or posterior fiducials are created by diagonal bars, they increase the area of the polygon enclosed by fiducials in the CT image and decrease the RMSe of the V-localizer compared to the RMSe of the Z-localizer [4,7,9,10]. The four colinear sets of fiducials have the further advantage that each set of fiducials is no more difficult to interpret for the four V-localizer configuration than for the two V-localizer configuration. In contrast, interpretation of the fiducial pattern for the Z-localizer is difficult [7].

The four V-localizer configuration creates two colinear sets of fiducials that lie in close proximity to one another near each of the left and right edges of the CT image, as shown in Figure 3B. It is imperative that each fiducial be assigned to the correct set instead of the adjacent set. The correct assignment may be facilitated via the calculation of a 2D linear least-squares correlation coefficient to measure the colinearity of the fiducials within a set upon tentative assignment of a fiducial to that set. A reviewer of this article has suggested that correct assignment would be facilitated if the fiducials of one set had a larger cross-section than the fiducials of the adjacent set. This viable suggestion could be implemented by enlarging the vertical and diagonal bars in the dimension perpendicular to the plane of the V-localizer.

**Figure 6:**
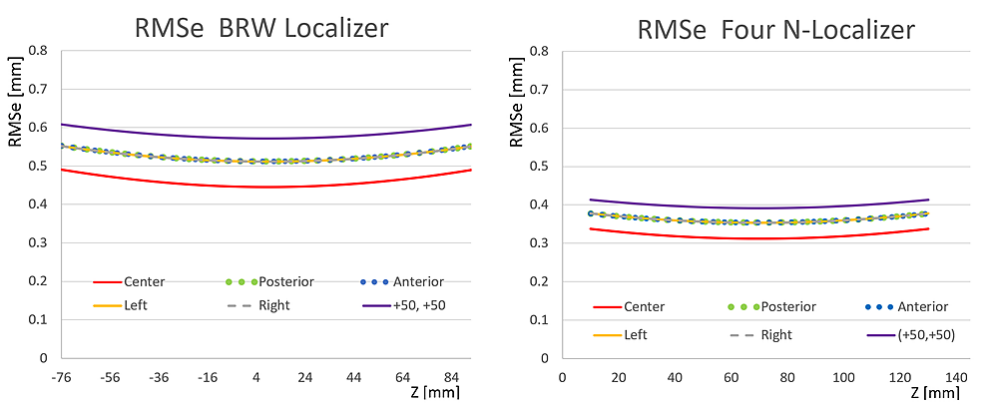
Plots of RMSe vs. Height $\left(Z\right)$ for the BRW Localizer and the Four N-Localizer Configuration The RMSe of the BRW localizer exceeds the RMSe of the four N-localizer configuration for each target point at all heights $\left(z\right)$. For both localizers, the plots for anterior, posterior, left, and right targets are superimposed. RMSe: root mean square errors; BRW: Brown-Roberts-Wells

Figure 6 presents RMSe plots for comparison to Figures 4, 5. This figure includes RMSe plots for a four N-localizer configuration [4] and for the Brown-Roberts-Wells (BRW) localizer wherein three N-localizers are arranged in an optimum configuration that minimizes error [10]. Comparison of Figures 4, 5, 6 reveals that the RMSe of the BRW localizer is less than the RMSe of the M-localizer, greater than the RMSe of the two V-localizer configuration except for the anterolateral $\left(+50, +50\right)$ target point, and greater than the RMSe of the Z-localizer and the four V-localizer configuration. Comparison of Figures 4, 5, 6 also reveals that the RMSe of the four V-localizer configuration is less than the RMSe of all other localizers, except for the anterior and posterior target points of the four N-localizer configuration for which the RMSe equals the RMSe of the four V-localizer configuration.

**Figure 7:**
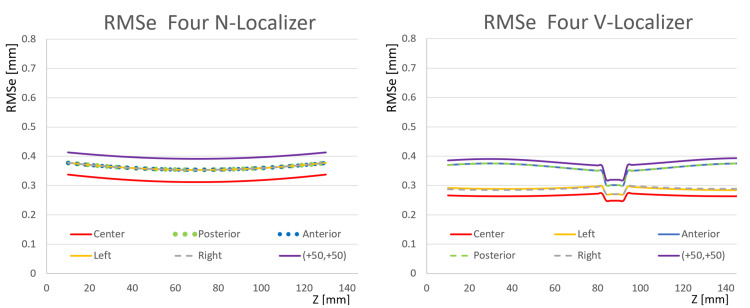
Plots of RMSe vs. Height $\left(Z\right)$ for the Four N-Localizer Configuration and the Four V-Localizer Configuration The RMSe of the four N-localizer configuration equals the RMSe of the four V-localizer configuration for the anterior and posterior target points, but exceeds the RMSe of the four V-localizer configuration for all other target points, at all heights $\left(z\right)$. For the four N-localizer configuration, the plots for anterior, posterior, left, and right targets are superimposed. For the four V-localizer configuration, the plots for anterior and posterior targets are superimposed and the plots for left and right targets are superimposed. The discontinuities of infinite slope in the plots for the V-localizer occur at heights where a diagonal bar abuts against a vertical bar. RMSe: root mean square errors

Figure 7 facilitates the comparison of the RMSe of the four N-localizer configuration to the RMSe of the four V-localizer configuration shown in Figure 2B. The RMSe of the four N-localizer configuration equals the RMSe of the four V-localizer configuration for the anterior and posterior target points but exceeds the RMSe of the four V-localizer configuration for all other target points. Although the four N-localizer configuration achieves high accuracy [4], the four V-localizer configuration achieves even higher accuracy, and hence is the most accurate localizer proposed to date.

The BRW localizer and the four N-localizer configuration have two advantages relative to the M-localizer, Z-localizer, and V-localizer. First, because the three N-localizers of the BRW localizer are positioned at $120^{\large\circ}$ intervals around the patient's cranium, they surround the cranium symmetrically. Similarly, the N-localizers of the four N-localizer configuration are positioned at $90^{\large\circ}$ intervals around the patient's cranium, so they also surround the cranium symmetrically. Hence, neither the BRW localizer nor the four N-localizer configuration is more susceptible to error anteriorly and posteriorly than laterally. Second, for either the BRW localizer or the four N-localizer configuration, each of the N-localizers creates in the CT image a set of only three colinear fiducials. These sets are not proximate geometrically, so unambiguous assignment of each fiducial to the correct set is simple.

In contrast to the four N-localizer configuration, the BRW localizer shares an important feature with the M-localizer, Z-localizer, and V-localizer. None of its three N-localizers obscure the patient's face; hence, claustrophobia is eliminated and patient comfort is maintained. In contrast to the BRW localizer and the four N-localizer configuration, none of the M-localizer, Z-localizer, or V-localizer surround the patient's cranium; hence, all of them are more susceptible to error anteriorly and posteriorly than laterally. Moreover, with the possible exception of the M-localizer, they lack the simplicity of the N-localizer.

## Conclusions

The M-localizer, Z-localizer, and V-localizer are all designed for attachment to the two lateral faces of a rectangular stereotactic frame. These localizers all create sufficient fiducials in a CT image to determine the three-dimensional spatial orientation of the CT image plane relative to the stereotactic frame. With the possible exception of the M-localizer, they create more complex fiducial patterns than do the four N-localizer configuration and the BRW localizer.

Monte Carlo simulations have calculated the RMSe for the M-localizer, Z-localizer, BRW localizer, two and four V-localizer configurations, and four N-localizer configuration. The simulations predict the following comparative accuracies for these localizers. The M-localizer, Z-localizer, and V-localizer are less accurate anteriorly and posteriorly than laterally. The accuracies of the Z-localizer, BRW localizer, four N-localizer configuration, and four V-localizer configuration are symmetric side-to-side but the accuracies of the two V-localizer configuration and the M-localizer are asymmetric side-to-side. The two V-localizer configuration and the BRW localizer are more accurate than the M-localizer. The Z-localizer and both V-localizer configurations are more accurate than the BRW localizer. The four V-localizer configuration is more accurate than the four N-localizer configuration and hence is the most accurate of all localizers.

## Figures and Tables

**Figure 8:**
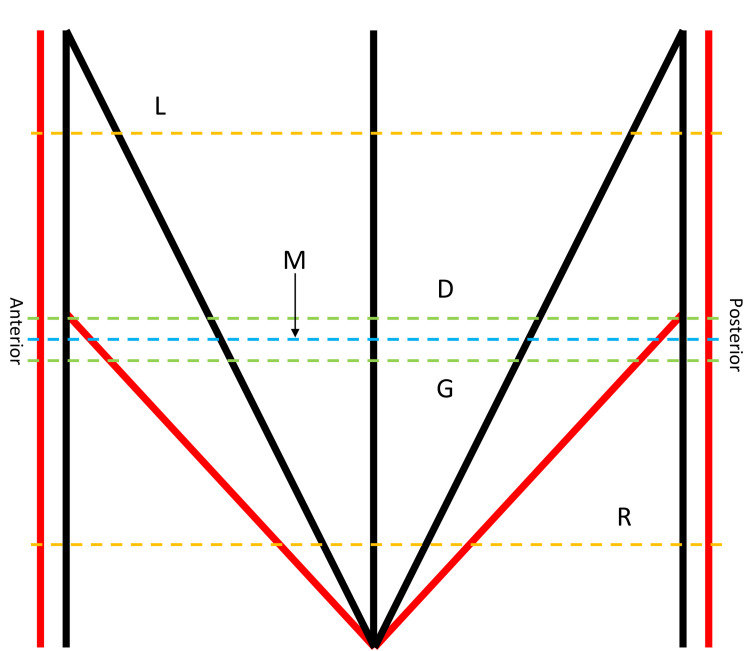
Depiction of the V-Localizer The black lines depict M-localizer elements. The red lines depict Z-localizer elements. The red diagonal lines do not span the entire height of the V-localizer but instead abut against black vertical lines. The dashed lines R, L, D, G, and M represent intersections of CT scan slices with the V-localizer. Dashed lines of the same color represent intersection with only one CT scan slice. Compare to Figure 1.

**Figure 9:**
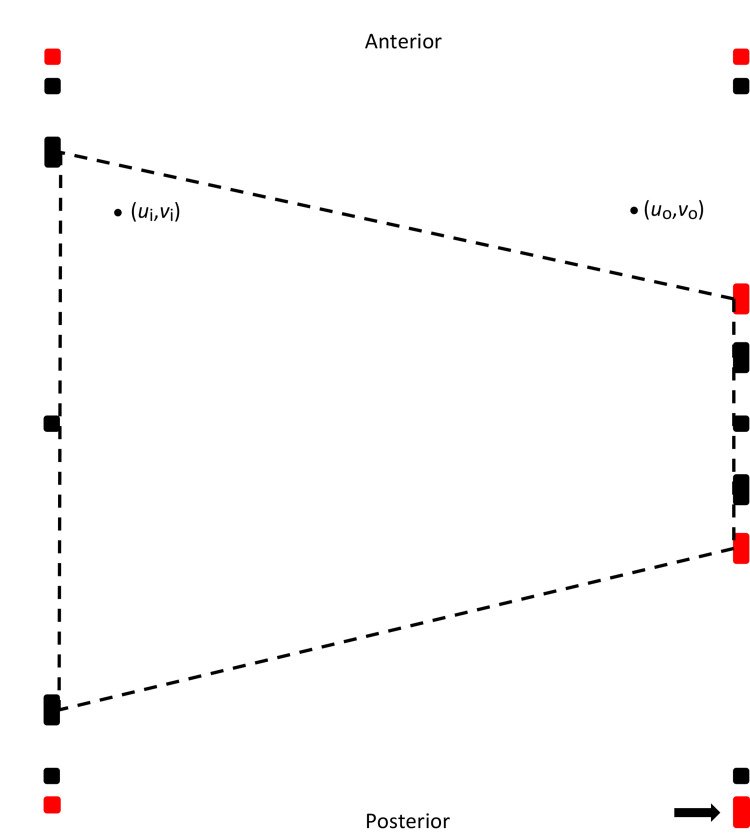
Intersection of a CT Scan Slice With Two V-Localizers The intersection of a CT scan slice with the two V-localizer configuration shown in Figure 2A creates fiducials in a CT image. The fiducials are colored according to the color of the corresponding bar in Figure 8. The black square fiducials are created by vertical bars of Figure 1 that correspond to black vertical bars of Figure 8. The black rectangular fiducials are created by $59.30^{\large\circ}$ diagonal bars of Figure 1 that correspond to black diagonal bars of Figure 8. The red rectangular fiducials are created by $42.81^{\large\circ}$ diagonal bars of Figure 1 that correspond to red diagonal bars of Figure 8. The sizes of the rectangular fiducials are not depicted accurately in this Figure 9. Due to the partial volume effect [11, 12], a high-slope $\left(59.30^{\large\circ}\right)$ diagonal bar creates a shorter rectangular fiducial than does a low-slope $\left(42.81^{\large\circ}\right)$ diagonal bar. Hence, the black rectangular fiducials should be shorter than the red rectangular fiducials, as represented in Figure 3. The rectangular fiducials that correspond to diagonal bars form a polygon depicted by black dashed lines in the $\left(u,v\right)$ coordinate system of the CT image. The point $\left(u_i,v_i\right)$ lies inside the polygon and the point $\left(u_o,v_o\right)$ lies outside the polygon. The red rectangular fiducial designated by the arrow is created by an oversized vertical bar that facilitates unambiguous identification of the fiducials [13].

**Figure 10:**
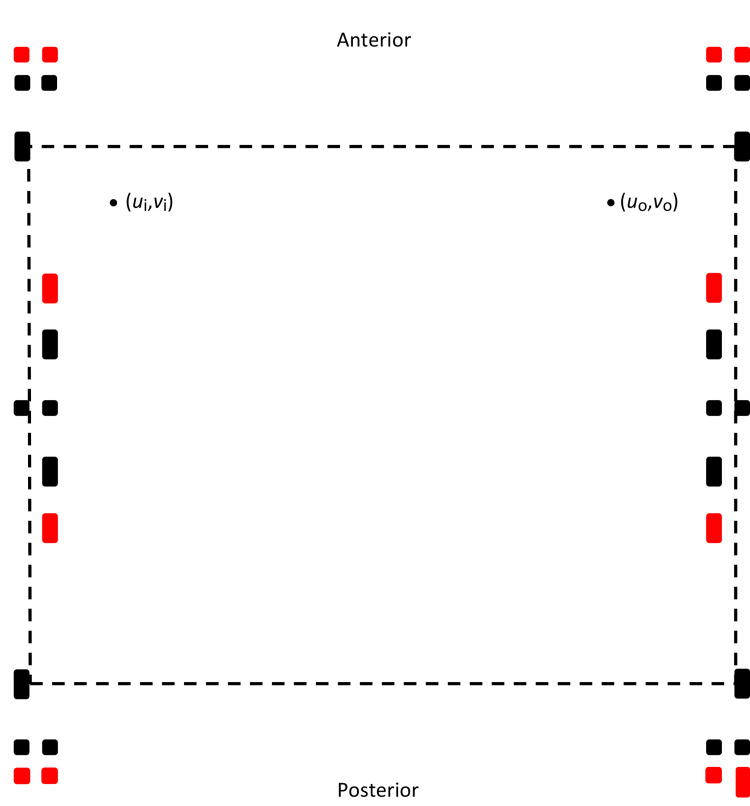
Intersection of a CT Scan Slice With Four V-Localizers The intersection of a CT scan slice with the four V-localizer configuration shown in Figure 2B creates fiducials in a CT image. The polygon formed by the farthest anterior and posterior diagonal fiducials is depicted by black dashed lines. This polygon encompasses both the point $\left(u_i,v_i\right)$ and the point $\left(u_o,v_o\right)$. Compare to Figure 3B.

**Figure 11:**
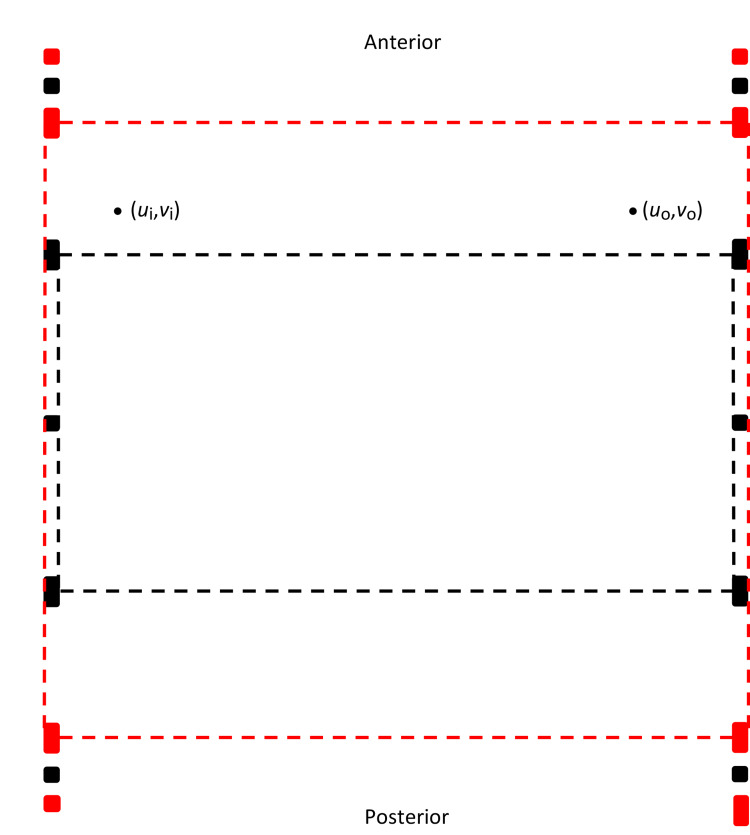
Intersection of a CT Scan Slice With Two V-Localizers at Mid-height (Mid-$Z$) The intersection of a CT scan slice at mid-height (mid-$z$) with the two V-localizer configuration shown in Figure 2A creates fiducials in a CT image. The vertices of the polygon depicted by black dashed lines are fiducials created by the black diagonal bars of Figure 8 that are elements of the M-localizer. The vertices of the polygon depicted by red dashed lines are fiducials created by the red diagonal bars of Figure 8 that are elements of the V-localizer. The red polygon encloses the points $\left(u_i,v_i\right)$ and $\left(u_o,v_o\right)$ but the black polygon does not enclose them. Compare to Figure 3A.

**Figure 12:**
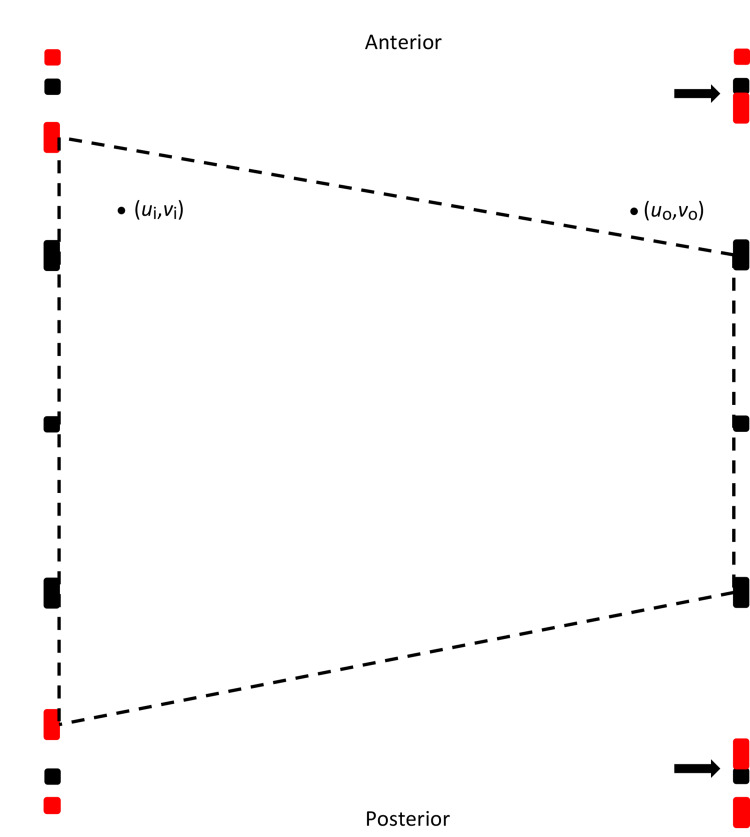
Intersection of a CT Scan Slice With One V-Localizer at an Unfavorable Height $\left(Z\right)$ The intersection of a CT scan slice with the two V-localizer configuration shown in Figure 2A creates merged fiducials in a CT image. The CT scan slice intersects the non-inverted V-localizer at the height where red diagonal bars abut against black vertical bars and hence creates the merged fiducials designated by arrows near the right edge of the CT image. The CT scan slice does not intersect the inverted V-localizer at the height where red diagonal bars abut against black vertical bars and hence the fiducials near the left edge of the CT image are not merged.

**Figure 13:**
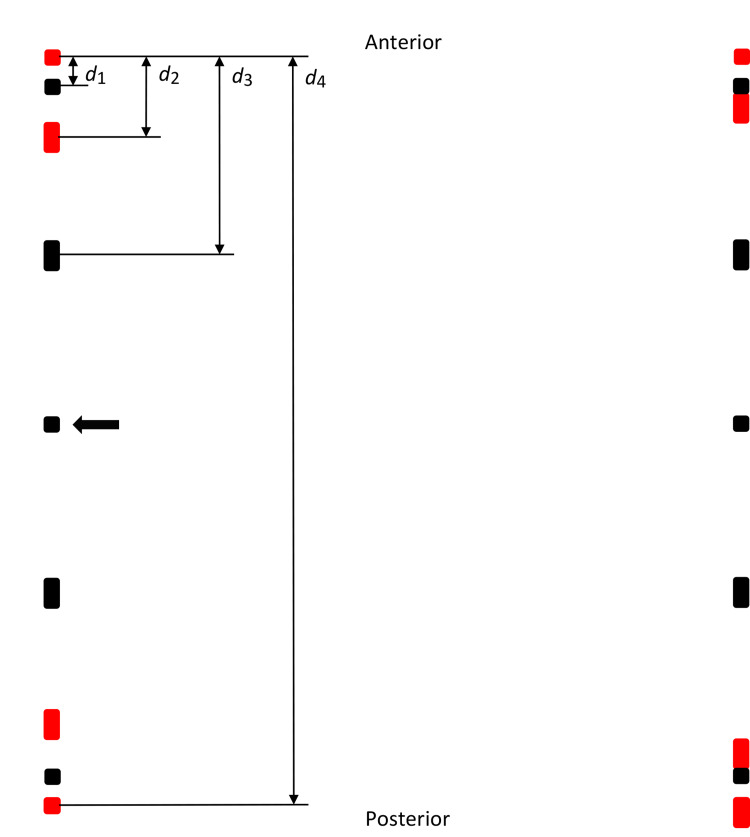
Filtering Each Fiducial by Its N-Localizer Interpolant The N-localizer interpolant $f_1=d_1/d_4$ is a constant that may alternately be calculated as $f_C=10/210\approx0.05$ directly from the geometry of the V-localizer depicted in Figure 1. The N-localizer interpolants $f_1=d_1/d_4$, $f_2=d_2/d_4$, and $f_3=d_3/d_4$ are compared to the constant interpolant $f_C$ to determine the eligibility of their respective fiducials for use in transforming a target point from 2D to 3D.

## Appendices

Detailed design of the V-localizer

Figure 8 depicts the V-localizer that combines elements from the M-localizer and Z-localizer [7]. The black lines (or bars) depict elements from the M-localizer. The red lines (or bars) depict elements from the Z-localizer. The black diagonal bars and all vertical bars span the entire height of the V-localizer. The red diagonal bars do not span the entire height of the V-localizer but instead abut against black vertical bars at a height $\left(z\right)$ that is slightly above mid-height. The dashed lines R, L, D, G, and M represent intersections of CT scan slices with the V-localizer. Dashed lines of the same color represent intersection with only one CT scan slice. For example, the orange dashed line R (right) represents the intersection of a CT scan slice with the non-inverted V-localizer affixed to the right face of the frame shown in Figure 2A and the orange dashed line L (left) represents the intersection of the same CT scan slice with the inverted V-localizer affixed to the left face of that frame. Similarly, the green dashed line D (from French “*droite*”, meaning “right”) represents the intersection of a CT scan slice with the non-inverted V-localizer affixed to the right face of the frame and the green dashed line G (from French “*gauche*”, meaning “left”) represents the intersection of the same CT scan slice with the inverted V-localizer affixed to the left face of the frame. There is only one blue dashed line M (mid-$z$) because the CT scan slice intersects both the non-inverted V-localizer and the inverted V-localizer at the same height $\left(z\right)$. Each of these intersections is discussed in detail below.

For the two V-localizer configuration shown in Figure 2A, one V-localizer is affixed to each lateral face of the frame. Each V-localizer is inverted top-to-bottom relative to the V-localizer that is affixed to the opposite face of the frame, similar to the configuration proposed for two M-localizers [7]. The V-localizer affixed to the right face of the frame is upright, as depicted in Figure 8, and the V-localizer affixed to the left face of the frame is inverted top-to-bottom. The intersection of a CT scan slice with the V-localizer affixed to the right face of the frame is depicted by the orange dashed line R in Figure 8. The intersection of the same CT scan slice with the inverted V-localizer affixed to the left face of the frame is depicted by the orange dashed line L in Figure 8. The CT image created by the intersection of this CT scan slice with both V-localizers is depicted in Figure 9. Each bar of the V-localizer creates a fiducial in the CT image. These fiducials are used to transform the $\left(u, v\right)$ coordinates of a target point from the 2D coordinate system of the CT image into $\left(x, y, z\right)$ coordinates in the 3D coordinate system of the stereotactic frame [8]. In particular, the $\left(u, v\right)$ coordinates of the centroid of each fiducial that is created by a diagonal bar (aka a diagonal fiducial) correspond to the $\left(x, y, z\right)$ coordinates of a point that lies on the long axis of the diagonal bar. Previous studies have reported that if a target point lies inside the polygon whose vertices are the diagonal fiducials, the RMSe of the transformed target point is lower than if the target point lies outside that polygon [4,7,9,10].

The polygon shown in Figure 9 reveals a vulnerability that the V-localizer shares with the M-localizer: the polygon and hence the RMSe of the V-localizer exhibit side-to-side asymmetry [7]. The polygon has a short right edge for a CT image obtained near the bottom of the stereotactic frame shown in Figure 2A and it has a short left edge for a CT image obtained near the top of that frame. Hence, the RMSe is higher on the right side of a CT image obtained near the bottom of the frame and the RMSe is higher on the left side of a CT image obtained near the top of the frame, as shown in Figure 4.

The four V-localizer configuration shown in Figure 2B eliminates the V-localizer asymmetry by affixing four V-localizers to the stereotactic frame. Two parallel V-localizers are affixed to each lateral face of the frame. One of the two V-localizers is inverted top-to-bottom relative to the adjacent V-localizer. The CT image created by the intersection of the CT scan slice depicted by the orange dashed lines R and L of Figure 8 with the four V-localizer configuration shown in Figure 2B is depicted in Figure 10. The polygon whose vertices are the farthest anterior and posterior diagonal fiducials is symmetric side-to-side, so the RMSe exhibits side-to-side symmetry, as shown in Figure 5. The point $\left(u_o,v_o\right)$ that lies outside the polygon of Figure 9 lies inside the polygon of Figure 10. Hence, the transformed $\left(x,y,z\right)$ coordinates of this point will have lower RMSe for the four V-localizer configuration shown in Figure 2B than for the two V-localizer configuration shown in Figure 2A. This lower RMSe is demonstrated by Figures 4, 5.

The CT image depicted by Figure 11 demonstrates that the V-localizer mitigates a vulnerability of the M-localizer for which the RMSe is maximum at mid-height [7], as shown in Figure 4. The CT image is created by the intersection of the CT scan slice depicted by the blue dashed line M (at mid-height or mid-$z$) in Figure 8 with the two V-localizer configuration shown in Figure 2A. The fiducial vertices of the polygon depicted by black dashed lines are created by elements of the M-localizer. The fiducial vertices of the polygon depicted by red dashed lines are created by elements of the V-localizer. Both the point $\left(u_i,v_i\right)$ and the point $\left(u_o,v_o\right)$ lie outside the black polygon but inside the red polygon. Hence, the transformed $\left(x,y,z\right)$ coordinates of both points will have lower RMSe for the V-localizer than for the M-localizer, as shown in Figure 4. The M-localizer exhibits this vulnerability because the vertices of the black polygon for a mid-height CT image do not extend anteriorly and posteriorly to a sufficient extent to provide low RMSe for transformed target points from those regions of the CT image. The V-localizer mitigates this vulnerability by extending the vertices of its red polygon anteriorly and posteriorly relative to the vertices of the black polygon of the M-localizer.

Figure 12 reveals a vulnerability that the V-localizer shares with the Z-localizer: the red diagonal bars of Figure 8 do not span the entire height of the V-localizer [7]. Instead, each red diagonal bar abuts against a black vertical bar. A CT scan slice that intersects such an abutment is depicted by the green dashed line D positioned slightly above mid-$z$ in Figure 8. The intersection of that CT scan slice with the non-inverted V-localizer affixed to the right lateral face of the stereotactic frame shown in Figure 2A creates fiducials near the right edge of the CT image shown in Figure 12. The arrows designate two red diagonal fiducials that have merged with adjacent black vertical fiducials. Because the $\left(u,v\right)$ coordinates of a fiducial are calculated as the centroid of all the pixels that the fiducial comprises, the $\left(u,v\right)$ coordinates of a merged fiducial represent the centroid of two fiducials taken together instead of the centroid of only one fiducial [7]. Hence, the $\left(u,v\right)$ coordinates of a merged fiducial are erroneous and cannot be used in the transformation of a target point from 2D to 3D because those erroneous coordinates would increase the RMSe [14].

Near the left edge of the CT image shown in Figure 12, the red diagonal fiducials have not merged with adjacent black vertical fiducials. The fiducials near the left edge of the image are created by the intersection of the CT scan slice depicted by the green dashed line G in Figure 8 with the inverted V-localizer affixed to the left lateral face of the stereotactic frame shown in Figure 2A. For an inverted V-localizer, the height where each red diagonal bar abuts against a black vertical bar is slightly below mid-$z$. Hence, a CT scan slice that intersects the abutments of the non-inverted V-localizer (green dashed line D) does not intersect the abutments of the inverted V-localizer (green dashed line G). Indeed, the abutments are placed slightly below mid-$z$ for an inverted V-localizer (or equivalently, slightly above mid-$z$ for a non-inverted V-localizer) to minimize the probability that any CT scan slice will intersect all four abutments, assuming that the CT scan slice is roughly perpendicular to the vertical bars. Intersection with all four abutments would cause each of the four red diagonal fiducials to merge with its adjacent black vertical fiducial and hence become unusable. In that case, the remaining usable fiducials would form the vertices of the polygon depicted by black dashed lines in Figure 11. The vertices of that polygon do not extend anteriorly and posteriorly to a sufficient extent to provide low RMSe for transformed target points from those regions of the CT image, as shown in Figure 4.

Given that a merged fiducial cannot be used in the transformation of a target point from 2D to 3D, a reliable method for identifying merged fiducials is essential to the proper operation of the V-localizer. One method filters a fiducial by its N-localizer interpolant. Interpolation for the N-localizer occurs along the long axis of a diagonal bar using a linear interpolant that is calculated from three fiducials [6,8]. The details of interpolant calculation have been published previously [1,3,6,8] and will be summarized below only briefly.

Figure 13 depicts a CT image that has the same pattern of fiducials as Figure 12. Near the left edge of the image, the red diagonal fiducials have not merged with adjacent black vertical fiducials. The method of filtering a fiducial by its N-localizer interpolant exploits the constant interpolant $f_C$ of a black vertical fiducial that corresponds to a black vertical bar against which a red diagonal bar abuts, as depicted in Figure 8. Typically, an N-localizer interpolant $f$ is calculated for a diagonal fiducial whose $\left(u,v\right)$ coordinates change according to the height $\left(z\right)$ of the CT scan slice; hence, $f$ is a linear function of $z$. For example, the interpolants $f_2$ and $f_3$, which are calculated as $f_2=d_2/d_4$ and $f_3=d_3/d_4$ for interpolation along red and black diagonal bars, respectively, change according to the height of the CT scan slice. Moreover, it is possible to calculate the N-localizer interpolant for a vertical fiducial even though that interpolant is constant and independent of the height of the CT scan slice even when that slice is not perpendicular to the vertical bars, consistent with the properties of similar triangles [15]. Hence, the interpolant $f_1$ for the black vertical fiducial may be calculated as $f_1=d_1/d_4$ but this constant interpolant may alternately be calculated from the V-localizer geometry presented in Figure 1 as $f_C=10/210\approx0.05$. This constant interpolant $f_C$ may be used to filter each fiducial by its N-localizer interpolant in the following manner. The interpolants $f_1$, $f_2$, and $f_3$ are calculated as $f_1=d_1/d_4$, $f_2=d_2/d_4$, and $f_3=d_3/d_4$ and then compared to the constant interpolant $f_C$. If any of these interpolants $f_i$ is sufficiently close to $f_C$, for example, $f_i \lessapprox 0.1$, the corresponding fiducial will be presumed to have merged with the black vertical fiducial. Consequently, that presumably merged fiducial will be excluded from the calculation of the transformation for a target point from 2D to 3D. This algorithm will always exclude the black vertical fiducial whose interpolant is $f_1$ and may exclude as well the red diagonal fiducial whose interpolant is $f_2$ but will never exclude the black diagonal fiducial whose interpolant is $f_3$ because the corresponding black diagonal bar does not abut against a black vertical bar. Although the simple depiction of Figure 8 shows that the black diagonal bar intersects a black vertical bar, Figure 1 reveals that the two bars do not in fact intersect.

Figure 13 and the above equations for the interpolants $f_1$, $f_2$, and $f_3$ demonstrate that these interpolants are calculated via division by the distance $d_4$ between two red vertical fiducials. In principle, the distance between one red vertical fiducial and the black vertical fiducial designated by the thick, single-ended arrow in Figure 13 could serve instead as the divisor [7]. However, the larger distance between two red vertical fiducials results in lower RMSe [3].
